# Development of tonality and consonance categorization ability and preferences in 4- to 6-year-old children

**DOI:** 10.3389/fpsyg.2024.1270114

**Published:** 2024-06-17

**Authors:** Johanna Karoline Will, Christina Roeske, Franziska Degé

**Affiliations:** Max Planck Society, Max Planck Institute for Empirical Aesthetics, Music Department, Frankfurt, Germany

**Keywords:** development, preference, perception, tonality, consonance

## Abstract

Consonance perception has been extensively studied in Western adults, but it is less clear how this perception develops in children during musical enculturation. We investigated how this development occurs in 4- to 6-year-old children by examining two complex musical skills (i.e., consonance and tonality preferences). Accordingly, we developed a child-focused approach to understand the underlying developmental processes of tonality and consonance preferences in 4- to 6-year-old children using a video interview format. As previous studies have confounded preference with perception, we examined each concept separately and measured perceptual abilities as categorization. For tonality, the ability to categorize tonal and atonal melodies developed by the age of 6 years. It is noteworthy that only children who could categorize successfully showed a preference for tonality at the age of 6. For consonance, we observed an early preference for consonance at 4 years of age, but this preference was only measurable with large differences between consonant and dissonant stimuli. We propose that tonality and consonance preferences develop during childhood with increasing categorization ability when the surrounding musical culture is marked by Western tonality and consonance.

## 1 Introduction

Consonance preference has long been thought to be innate, with studies suggesting that both infants (Schellenberg and Trehub, 1996; Trainor et al., 2002) and adults exposed to the Western music system prefer consonance to dissonance (Butler and Daston, 1968; McDermott et al., 2016). However, attempts to replicate the early preference findings have failed (Plantinga and Trehub, 2014), and studies with children suggest an influence of exposure. Consonance preference appears to have a biological basis (Bowling et al., 2017) and is influenced by age and musical experience (Valentine, 2015; Weiss et al., 2020). To date, how a so-called preference for consonant stimuli in infancy develops into Westerners’ strong preference for consonance in adulthood remains unknown. Furthermore, it is questionable whether this distinction is fruitful, or whether a more continuous view of nature vs. culture would be more appropriate (Di Stefano et al., 2017). Previous research on the development of tonality perception, a similarly complex musical concept, suggests that the study of enculturation as an explanatory mechanism at preschool age seems most promising for further research (Miyamoto, 2007). Therefore, we investigate tonality and consonance preference (two complex musical skills) in children between the ages of 4 and 6 (i.e., the preschool years) in order to compare and their development and understand it in more detail. In previous studies, the concepts of consonance perception and preference have been confounded; therefore, we distinguish between them using a new child-friendly paradigm.

As children develop, cultural and individual musical experiences shape their perceptions of music (Trehub et al., 2015), which shift from relying on culture-independent to culture-specific strategies (Trehub et al., 1997). With increasing age, children specialize in the music of their environment and switch to culture-specific strategies (Trehub et al., 1990; Lynch and Eilers, 1992). However, the point at which children begin to categorize at an adult level depends on the stimulus selection and their cultural experience. Children are generally better at categorizing sounds that are typical of the dominant surrounding musical culture (Matsunaga et al., 2020), whereas categorization of unfamiliar music remains at chance level (Lynch et al., 1990). Therefore, understanding the development of preferences is of particular interest for investigating how the ability to categorize between tonality and atonality or consonance and dissonance develops in children exposed to a Western music environment.

The concept of tonality, which refers to the connection of tones through their relationship to a common main tone within a piece of music (Helmholtz according to Cuddy, 1978), develops rather late in childhood, since the internalization of tonal rules is a complex perceptual task. Furthermore, the point at which children understand and act upon tonal rules remains unclear. In general, children’s music perception skills seem to improve during the primary school years (Boyle and Penticoff, 1989) and can be further developed with musical experience (Halpern et al., 1998; Corrigall and Trainor, 2009, 2010). With regard to tonality, it is known that tonal perception is still developing around the age of 5 and that the rules are not yet fully internalized (Schwarzer et al., 1993). However, a processing advantage for tonal melodies already exists, as changes in tonal melodies are recognized better than those in atonal melodies (Trehub et al., 1986). Between the ages of 5 and 10, children increasingly internalize the tonal rules of Western music (Maier-Karius and Schwarzer, 2011). Although 5- to 6-year-old children reportedly do not express a preference for the tonic as the ending of tonal melodies (Schwarzer et al., 1993; Maier-Karius and Schwarzer, 2011), 6- to 7-year-old children (Maier-Karius and Schwarzer, 2011), 9- to 10-year-old children (Schwarzer et al., 1993; Maier-Karius and Schwarzer, 2011), and adults (Schwarzer et al., 1993) do. Taken together, these studies suggest that enculturation into Western tonality is a late developing and complex pitch skill (Trainor and Hannon, 2013; Corrigall and Trainor, 2014) that occurs in childhood, and that knowledge of the implicit concept of the tonic affects musical perception as early as age 6 (Schellenberg et al., 2005). Thus, the early signs of tonality development at the age of 4, and how this compares with the development of other complex musical abilities, such as the perception of consonance and dissonance, seems to be an intriguing avenue of research. However, when interpreting and comparing these findings it is important to bear in mind that the method itself (i.e., asking about the end of a melody) is difficult to understand and may also underestimate children’s abilities.

The preference for consonance is a part of the tonal development in late childhood. In Western music, consonance is associated with smoothness and dissonance with roughness (Von Helmholtz, 1863). While consonance seems pleasant (Vassilakis, 2005) and satisfying in itself, it remains speculative why this is the case (Di Stefano et al., 2017). Dissonance is the opposite: Western listeners prefer dissonance to be resolved into consonance (Kennedy et al., 2012). Despite previous assumptions of a categorical difference between consonance and dissonance, current theoretical assumptions suggest that the degree of consonance decreases continuously toward dissonance as the complexity of frequency relations increases (Plomp and Levelt, 1965). However, in order to measure consonance preference and perception in childhood, our study is methodologically based on a categorical classification (which is easier for children) of consonance and dissonance, similar to a previous study by Plantinga and Trehub (2014).

Adults familiar with Western music showed better and faster detection of changes in consonant intervals (Schellenberg and Trehub, 1996) and advantages in processing consonance in terms of simple frequency ratios compared to their processing of dissonance (Schellenberg and Trehub, 1994). Beyond the processing advantages of consonance in adulthood, it has been repeatedly shown that adults prefer consonance (e.g., Weiss et al., 2020). Moreover, music training strengthens this preference (Weiss et al., 2020). In cross-cultural comparisons, however, the strong preference for consonance in adulthood is not universal. For example, non-Western listeners, such as the Tsimané of Bolivia, have been found to show no preference for either consonance or dissonance (McDermott et al., 2016), while Indians have shown a greater tolerance for dissonance than Westerners (Maher, 1976). This suggests that cultural factors could potentially influence responses to consonance and dissonance (McDermott and Oxenham, 2008; McDermott et al., 2010). Consistent with this, the effects of cultural exposure on consonance preference suggest that this preference is developing.

Precursors of consonance preference appear to be evident in infants and children. Western infants may already perceive consonance in a special way, with prolonged viewing of consonant stimuli in head-turn preference paradigms interpreted as indicating a preference for consonance over dissonance (Crowder et al., 1991; Schellenberg and Trehub, 1996; Schellenberg and Trainor, 1996a; Trainor and Heinmiller, 1998). In addition, easier processing for consonance (referred to as preference) has been found immediately after birth (Masataka, 2006), and sensitivity to consonance has been measured before knowledge of scale structure (Trainor et al., 2002). On a physiological level, infants show less motor activity and avoidance behavior when listening to consonance (Zentner and Kagan, 1996, 1998). However, prolonged listening to consonant stimuli in infancy has not been supported in an extensive replication study (Plantinga and Trehub, 2014). Consistent with this, initial findings show that the adult-like preference for consonance consolidates with age, at least in Western-socialized children (Valentine, 2015; Weiss et al., 2020). Overall, previous research has identified unconscious competencies for consonance and tonality that emerge very early in life and develop into preferences in adulthood. We undertook this study to investigate when these early competencies become conscious processes, focusing on preschool children to examine developmental changes in consonance preference in 4- to 6-year-old children and the underlying factors (e.g., categorization). We also developed a new method for assessing consonance preference to overcome problems of dissociating perception from preference raised by the extensive use of looking time measurements as a methodological approach (Harrison and Pearce, 2020). A similar endeavor for infants and younger children has been undertaken by Di Stefano et al. (2017). Using a musical toy, they demonstrated a preference for consonance in children aged 1.5–3 years. Without using a musical toy, we have disentangled these in our new method for older children.

### 1.1 The current study

We had three aims in this study. The first aim was to develop a new method to dissociate perception from preference. Inferring preference based on looking time alone can lead to inaccurate or erroneous information about music processing (Trehub, 2012). To overcome this conflation of preference and perception, we introduced an age-appropriate paradigm for children with separate tasks for preference and categorization. In Experiment 1, we investigated whether children can express their preferences (e.g., for food and drawings) and whether this paradigm is suitable for assessing categorization. The second aim was to explore the developmental trajectory of tonality preferences in 4- to 6-year-old children and how categorization develops. In Experiment 2, we used the same method as in Experiment 1, except that the musical stimuli were changed to tonal and atonal melodies. Our third aim, related to consonance, was to investigate when children categorize consonance and dissonance differently and at what point this is reflected at the action level, as in preference ratings. Therefore, in Experiment 3, we used a slight-difference-based condition in which tonal and atonal melodies (see Experiment 2) were accompanied by consonant harmonies. To increase the detectability of differences between stimuli, we added a large-difference-based condition, which consisted of tonal melodies with consonant harmonies and atonal melodies with dissonant harmonies. Cognitive theories, for example, embodied cognition (Shapiro and Spaulding, 2021) assume that cognitive tasks require effort in different amounts. Hence, our slight-difference-based condition varies in its cognitive load from the large-difference-based condition. The cognitive load of the large-difference-based condition is lower, because the task is easier to solve. Thus, this condition will potentially be solved successfully earlier in development compared to the slight-difference-based condition with its higher cognitive load. The stimuli in Experiments 2 and 3 were previously used by Plantinga and Trehub (2014). Due to contact restrictions during the COVID-19 pandemic, all experiments were conducted online. Different children participated in each experiment. Our study focuses on the tonality and consonance preferences of children who are regularly exposed to the Western music system; therefore, it cannot be assumed to be generalizable to other music systems.

## 2 Experiment 1: evaluation of the method

We developed a new method for testing children’s categorization abilities and preferences, which was evaluated in Experiment 1.

### 2.1 Method

#### 2.1.1 Participants

The final sample consisted of 48 (20 male and 28 female) children aged between 4 and 6 years (*M* = 66 months, *SD* = 11 months, 16 children per age group), who were recruited from kindergartens and through online advertisements. Cultural background was assessed based on country of birth and native language. All children in the sample were born in Germany and 91.70% of them spoke German as their native language. None of the children reported hearing impairments or color blindness.

Socioeconomic status was assessed based on parental education level and monthly family income. In most cases, neither parent had a university degree (37.50%), followed by those in which both parents had obtained a university degree (35.4%). Monthly family income ranged from 2,000 to 3,000 € (18.80%) to more than 5,000€ (31.30%). In the intermediate categories, the second most frequently reported was 4,000–5,000€ (29.20%), followed by 3,000–4,000€ (20.70%). On average, the children listened to music at home for 9 h per week (*SD* = 8 h). Two-thirds of the parents indicated that they made music (i.e., singing together or playing instruments) with their child every week (*M* = 3 days, *SD* = 2 days). The average duration of music lessons received was *M* = 10 months (*SD* = 14 months). About half of the children had at least one parent that had received a musical education (59.40%). Participating children took part in extracurricular activities for an average of *M* = 24 months (*SD* = 22 months). Participants received a certificate and could choose between 10€ or a 10€ book voucher as compensation. Data were collected in 2020, and our study was approved by the ethics commission of the Max Planck Society (request: 2019_23). From the original sample four participants’ data were excluded due to technical issues (*n* = 2) or disruptions in their home environments (*n* = 2). This resulted in the final sample size reported above.

To estimate the sample size, we conducted an *a priori* power analysis using G*Power version 3.1.9.4 (Faul et al., 2007, 2009). As we applied a new methodological approach in this experiment, we chose Cohen’s *d* = 0.8, because other studies applying preference measurement in childhood (e.g., Weiss et al., 2020) obtained similar effect sizes based on Cohen (1988). For comparability, this corresponds to *r* = 0.37, which is used as to measure effect size in this study. The minimum required sample size at a significance criterion of α = 0.05 and a power *(1-ß)* = 0.95 was *N* = 24 for Wilcoxon signed-rank tests. Therefore, our sample (*N* = 48) is more than adequate to evaluate the new methodological approach.

#### 2.1.2 Apparatus

**Figure 1:**
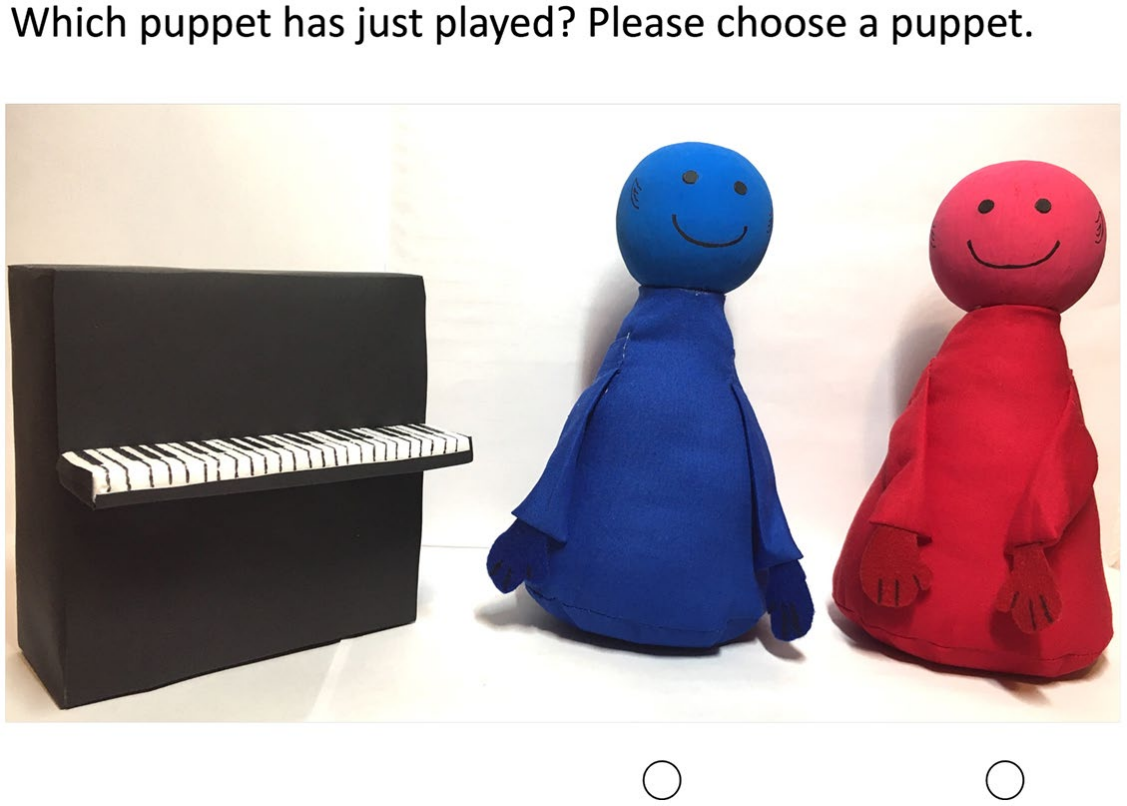
Screenshot of a video session for the assignment task to measure categorization ability. The children pointed to one of the dots under the puppets to indicate which puppet they thought had played the piano piece they had heard.

Testing took place individually at the participants’ own homes via Cisco WebEx video calls (Figure 1). Musical stimuli were created with MuseScore, transformed into mp3 audio files, and embedded into a PowerPoint file used for stimulus presentation. As the experimenter and participant were not in the same location, all stimuli were presented via WebEx’s screen-sharing function, with optimized settings for visual and auditory output.

#### 2.1.3 Stimuli

**Figure 2:**
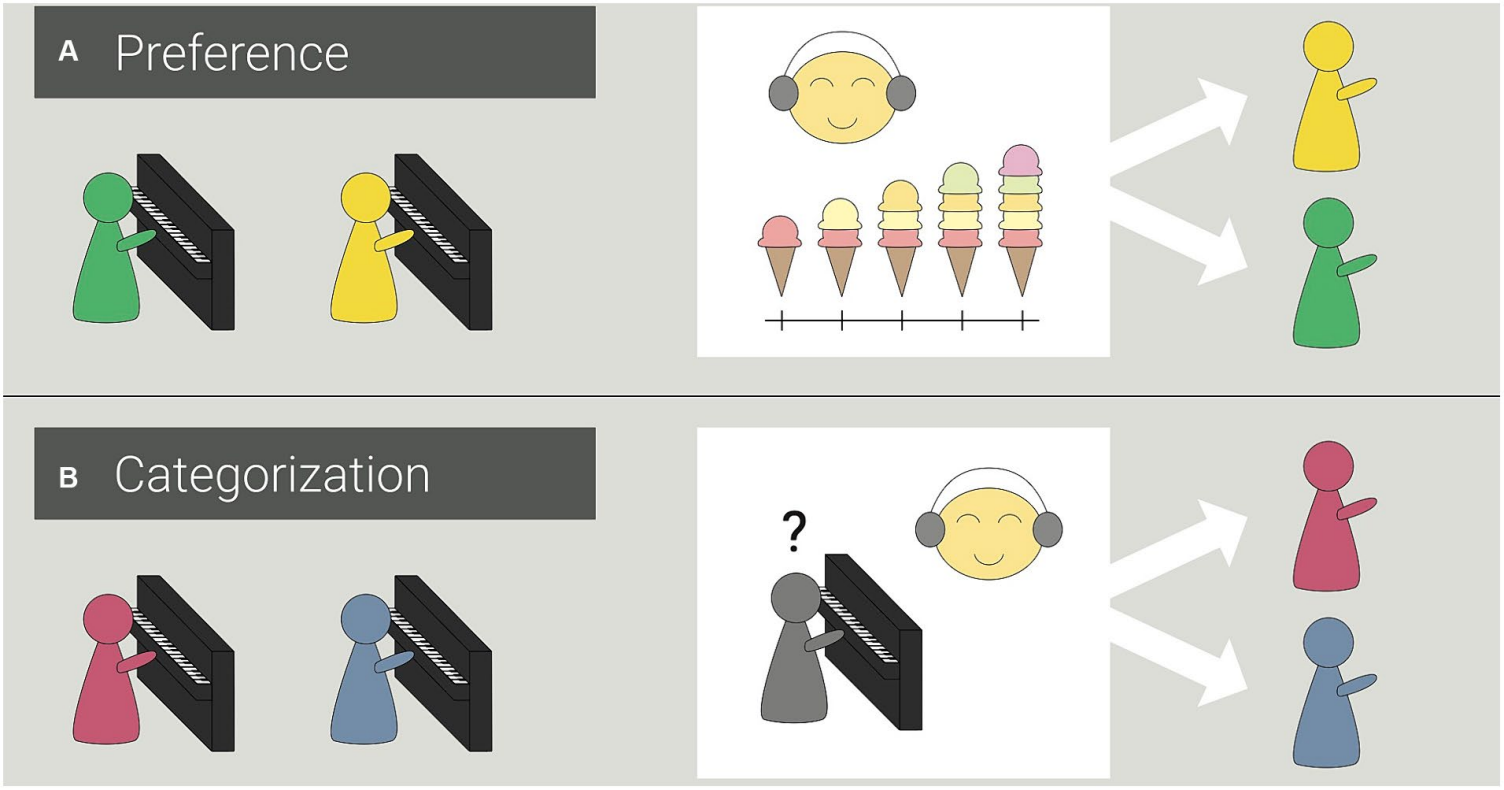
Procedure for measuring preferences **(A)** and categorization **(B)** in Experiments 1–3. **(A)** In Experiment 1, for evaluation purposes, children rated different food items and drawings using the ice cream scale in the preference task. Stimuli were replaced by piano pieces in Experiments 2 and 3. Children rated how much they liked the piano piece by rewarding puppets for their performance with ice cream. **(B)** To measure children’s categorization, children listened to two different piano pieces played by puppets in a training round. Afterward, a puppet whose color could not be seen played a piano piece. Children then assigned one of the puppets from the training round to the piece they assumed the puppet had just played on the piano.

Puppets (Figure 2), a toy piano, and a five-point scale with pictures of ice cream scoops representing scale points were used for age-appropriate visualization. Following Einarson et al. (2012), the puppets were positioned behind the toy piano to appear as if they were producing the musical stimuli themselves (Einarson and Trainor, 2015, 2016). The order of puppet pairs was balanced; however, the color pairs red–blue and yellow–green remained together in all tasks (Einarson and Trainor, 2015, 2016). In addition, the order within the pairs was balanced across participants (i.e., red–blue and yellow–green vs. blue–red and green–yellow). Puppets of different colors were used to provide a way for children to distinguish between the puppets when rating their preferences. As the participants should rate drawings and in the following experiments piano pieces played by the puppets, we decided to use puppets as actors to minimize any influences of liking. For the puppets the only difference between them is the color and we assessed the color preferences of the participants. Furthermore, we expected influences of social pressure (to give socially desirable answers) that might be strong for adult performances to be less relevant for puppet performances (please see limitations for potential problems with using puppets in developmental research; Packer and Moreno-Dulcey, 2022; Yu and Wellman, 2022).

#### 2.1.4 Data generation

Participants used pictures of ice cream to rate different foods (broccoli, carrots, chocolate, and chips) and drawings of two puppets to measure preference strength, The more the children liked to eat the food shown, the more scoops of ice cream they selected on the five-point scale (see Figure 2). The meaning of the amounts of ice cream was explained separately from the rest of the task to avoid tendencies toward extreme ratings (Chambers and Johnston, 2002). The key was defined as follows (German original text in parenthesis): “not at all (gar nicht)” (1 scoop), “a little (ein wenig)” (2 scoops), “neutral (unentschieden)” (3 scoops), “quite a bit (ziemlich viel)” (4 scoops), or “very much (sehr viel)” (5 scoops). To test whether 4- to 6-year-old children could use the scale to rate stimuli ostensibly produced by puppets, children rated two rainbow drawings that differed in quality: a symmetrical rainbow with clear demarcation between individual colors and an asymmetrical rainbow without demarcation between the individual colors, which looked more like a scribble. The participants were asked to rate the puppets’ drawings using scoops of ice cream as described above. The order of the drawings was balanced across participants.

During our categorization task, the puppets played two different piano pieces: the song “All My Little Ducklings” or wild key combinations that sounded like the puppet was hitting the keys at random. We used MuseScore to create all stimuli, which were matched in tempo (150 bpm) and length. Each piano piece was 16 s in duration, and the order of stimuli was balanced across participants. Overall, the order of preference and categorization measurement was also balanced across participants.

All control variables were assessed using parent interviews. Socioeconomic status was measured in terms of parental education and family income. Parental education was coded with 0 for *no parent with a university degree*, 1 for *at least one parent with a university degree*, and 2 for *both parents with university degrees*. Monthly income was rated on the following six-point scale: 1 for *under 1,000€*, 2 for *1,000–2,000€*, 3 for *2,000–3,000€*, 4 for *3,000–4,000€*, 5 for *4,000–5,000€*, and 6 for *over 5,000€*. Children’s musical experience was measured in terms of three aspects (music listening, joint music-making, and music lessons), with possible responses being “yes” or “no.” If parents responded with “yes,” they were asked for further information, including the amount of time (hours per week) that their child listened to music, how often they made music together with their child (days per week), and total duration of their child’s music lessons (months). All data were analyzed using IBM SPSS Statistics version 27 (IBM Corp., 2020) and R version 2022.07.2 (R Studio Team, 2022).

#### 2.1.5 Procedure

Children were seated in front of their computers, with their parents in the background (i.e., outside the child’s visual field). Parents were told to maintain a neutral facial expression and not influence their child’s decisions. For standardization purposes, all children were tested by the same investigator. She was previously trained to maintain a neutral facial expression during stimulus presentation and had no prior knowledge of the order of stimuli. Strength of preference was measured using a five-point scale (ice cream scoops, Figure 2). Beforehand, the scale was explained step by step, and the meanings were summarized (*“Choose more ice cream the more you like the food/drawing.”*). Children viewed pictures of different food items (broccoli, carrots, chocolate, and potato chips) and were asked “*How much do you like the food? Tell me your answer on the ice cream scale*.” Children rated how much they liked to eat each food item. Next, they were asked to rate how much they liked the drawings by the puppets. Categorization was tested using an assignment paradigm. Children were asked to match puppets and piano pieces, in response to the question *“Which puppet do you think just played this piano piece?”* The categorization task started with one training item, in which the puppets were visible while playing, before two experimental rounds. Here, the participants could learn which puppet (distinguishing by color) played each piece. In the first experimental round, the two songs were in the same order as that in the training item. In the second round, the order in which the piano pieces were presented was changed to control for sequence effects. In both experimental rounds, the puppets were not recognizable when playing the piano (see Figure 2). This allowed the child to answer the question of which puppet played the song without knowing for certain which color puppet it was. Finally, parents were interviewed regarding musical experience, socioeconomic status, cultural background, age, and gender, which served as control variables. Participation lasted 30 min in total. Children finished their portion in the first 15 min and could decide for themselves whether they wanted to stay for the parent interview.

### 2.2 Results

Experiment 1 was conducted to evaluate the new assessment methods for preferences and categorization. We evaluated the use of the rating scale with Wilcoxon signed-rank tests. The assignment paradigm was tested with Chi-Square tests. We conducted age group comparisons and examined the distribution of correct and incorrect answers.

#### 2.2.1 Using the rating scale

**Figure 3:**
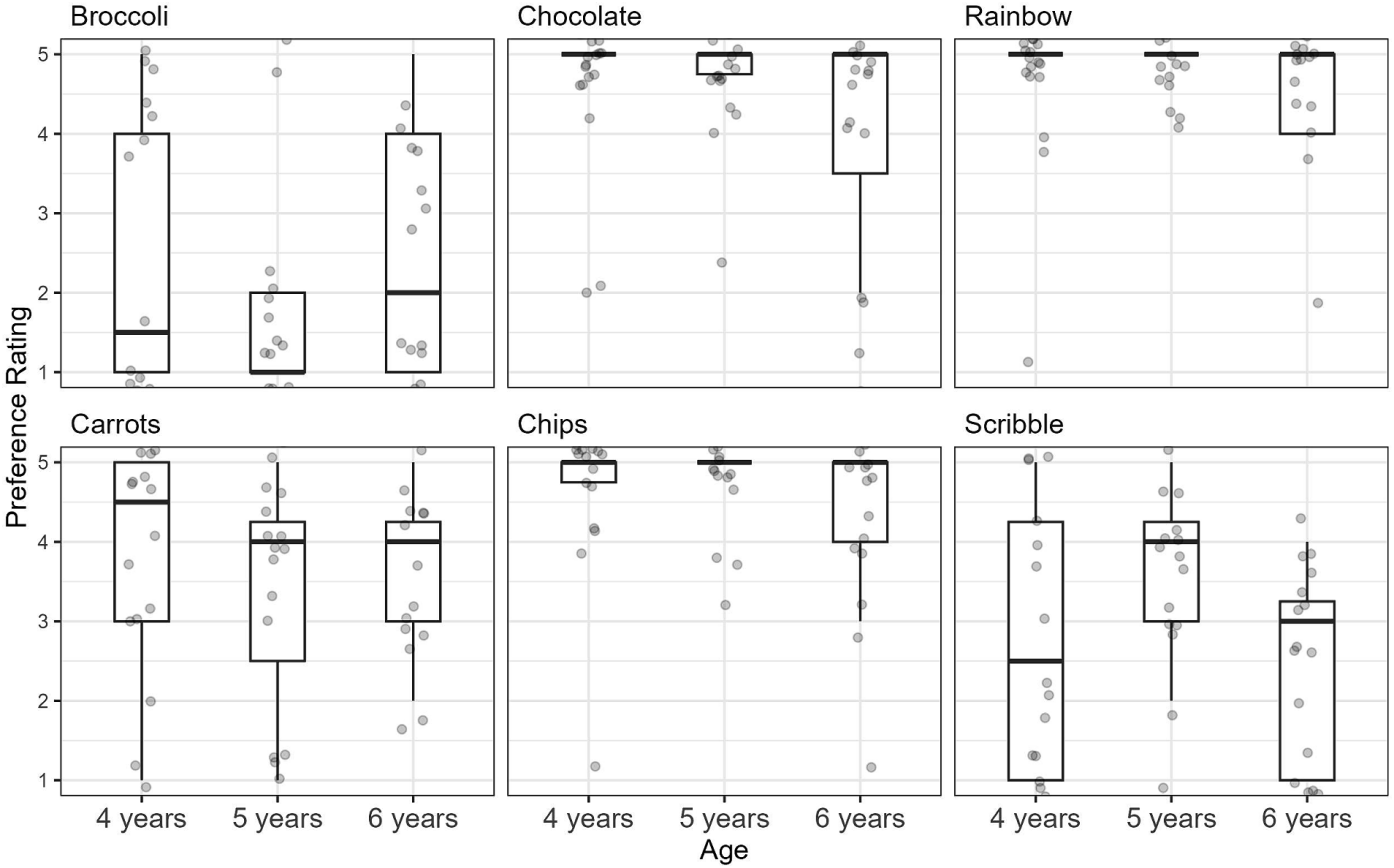
Mean preference ratings and SD in scoops of ice cream over all stimuli.

The five-point ice cream scale was used to measure children’s preferences for food and drawings. Figure 3 shows the means and standard deviations for preference ratings across age groups.

Wilcoxon signed-rank tests were conducted to determine whether 4- to 6-year-old children were able to use the scale to indicate their preferences. Table 1 summarizes all the comparisons performed.^1^ As expected, 4-year-old children showed a slight preference for carrots, and a greater preference for both chocolate and chips over broccoli. Correspondingly, 5-year-old children liked carrots a little more, chips even more, and chocolate most significantly more than broccoli. Overall, 5-year-old children liked sweets significantly more than healthy vegetables. The trend of not liking broccoli continued among 6-year-old children, who preferred chocolate a little, carrots somewhat more, and chips the most compared to broccoli.

**Table 1:** Wilcoxon signed-rank tests for food preferences across age groups.

Food	Age											
	4 years				5 years				6 years			
	*z*	*p*	*r*	*95% CI*	*z*	*p*	*r*	*95% CI*	*z*	*p*	*r*	*95% CI*
Broccoli against												
Carrots	−1.50	0.038	0.51	[−3.49, −0.00]	−2.29	0.043	0.51	[−3.00, −0.00]	−1.50	0.005	0.69	[−2.50, −0.50]
Chips	−2.50	0.004	0.74	[−4.00, −1.49]	−3.49	0.002	0.73	[−4.00, −1.49]	−2.49	0.004	0.71	[−3.50, −1.00]
Chocolate	−2.50	0.005	0.74	[−4.00, −1.00]	−3.38	0.001	0.80	[−4.00, −1.50]	−2.49	0.031	0.55	[−3.50, −1.50]

In evaluating the drawings in which the puppets’ artistic abilities were compared, children of all ages were able to express their preferences using the ice cream scale. Consequently, in Table 2, a significant preference for the beautiful drawing of a rainbow over the scribble occurred in 4-year-old, 5-year-old, and 6-year-old children.

**Table 2:** Wilcoxon signed-rank tests for artistic preferences across age groups.

Art	Age											
	4 years				5 years				6 years			
	*z*	*p*	*r*	*95% CI*	*z*	*p*	*r*	*95% CI*	*z*	*p*	*r*	*95% CI*
Drawing against												
Scribble	−2.49	0.017	0.63	[−3.49, −0.99]	−1.49	0.003	0.76	[−1.99, −0.99]	−2.00	<0.001	0.88	[−2.99, −1.49]

#### 2.2.2 Using the assignment paradigm

**Figure 4:**
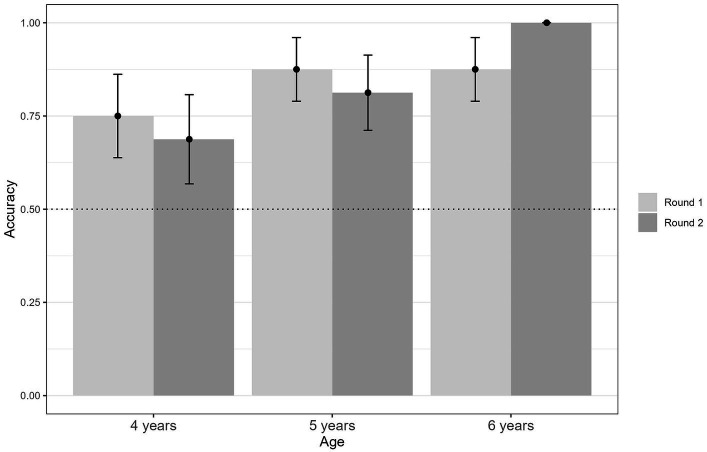
Categorization accuracy in 4- to 6-year-old children. Categorization accuracy improves between 4 and 6 years of age. When matching differently colored puppets to the song “All My Little Ducklings” or the wild piano key combinations, all age groups performed above chance (dotted line). Categorization reached significance at 5 years and improved with increasing age.

To measure categorization, all children completed two rounds in the assignment paradigm to eliminate sequence effects. Binomial distribution was used to test the quality of categorization based on whether significant positive deviations from chance were present (Figure 4). The categorization of 4-year-old children was already approaching significance for above chance assignments in Round 1 (*p* = 0.077, *n* = 16) and Round 2 (*p* = 0.210, *n* = 16), but not significantly. This changed at 5 years of age when the children’s categorization began to be significantly above chance in Round 1 (*p* = 0.004, *n* = 16) and Round 2 (*p* = 0.021, *n* = 16). In 6-year-old children, categorization improved even more, which was evident in Round 1 (*p* = 0.004, *n* = 16) and Round 2 (*p* < 0.001, *n* = 16). Overall, our results indicate a developmental trajectory in which categorization ability increases with age.

Response frequencies of correct and false assignments were compared between age groups to further assess the developmental trajectory of categorization, which could be observed in the binomial distribution tests. Chi-square tests were conducted to examine differences in response frequencies between age groups, and the results supported our previous results. Consistent with the binomial distribution test, correct assignments were significantly higher at age 6 (93.75%) than at age 4 (71.87%), *X*^2^ (1, *N* = 64) = 5.38, *p* = 0.020. This effect was reduced as the age gap decreased, as 6-year-old children (93.75%) could assign the correct piano pieces marginally significantly more often than 5-year-old children (83.38%), *X*^2^ (1, *N* = 64) = 3.14, *p* = 0.077. No significant differences were found in response frequencies between 4-year-old and 5-year-old children, *X*^2^ (1, *N* = 64) = 1.46, *p* = 0.226.

### 2.3 Discussion

Given the lack of a methodological approach to study preference and perception separately in children, two new tasks were developed and evaluated in Experiment 1. Children rated their preferences for food and drawings using a five-point scale depicting ice cream scoops. An assignment paradigm was used to test their perceptions of differences between musical stimuli. Overall, 4- to 6-year-old children were able to indicate their preferences using the child-friendly visualized scale. An evaluation of the assignment paradigm revealed a developmental change as age increased. Specifically, 5- and 6-year-old children explicitly categorized the musical stimuli at a level above chance and were thus able to accurately perform the categorization task. Age group comparisons showed increasing correct assignments with age. Taken together, the results indicate that all age groups were able to use the scale for preference ratings, and the assignment paradigm was successfully used to measure perception separately. Successful evaluation of the newly developed methodological opportunity allows both tasks to be used in the following experiments to investigate the development of tonality (Experiment 2) and consonance (Experiment 3) preferences and perceptions in 4- to 6-year-old children.

## 3 Experiment 2: development of tonality preference and categorization

Experiment 2 was conducted to measure the developmental trajectory of tonality preference and categorization using the method presented in Experiment 1. Therefore, we chose tonal and atonal melodies from Plantinga and Trehub (2014) and aimed for replication with an extension of the age group to 4- to 6-year-old children.

### 3.1 Method

#### 3.1.1 Participants

The final sample included 55 children (29 female) 4 to 6 years of age (*M* = 67 months, *SD* = 10 months). The age groups included *n* = 17 4-year-old children, *n* = 19 5-year-old children, and *n* = 19 6-year-old children, respectively. Recruitment and rewards for participation were the same as in Experiment 1. For most participants, both parents had obtained a university degree (58.2%), while 7.3% had neither parent with a university degree and 34.5% had one parent with a university degree. Monthly family income ranged from 1,000–2,000€ (3.6%) to more than 5,000€ (23.6%). Most parents specified their monthly income as between 4,000–5,000€ (27.3%) and 3,000–4,000€ (27.3%), followed by 2,000–3,000€ (18.2%). All children except one were born in Germany, and 83.6% spoke German as their native language. In their home environment, children listened to music 10 h per week on average, but variability was high (*SD* = 10 h). Among the parents, 87.3% indicated that they made music with their child every week (*M* = 3 days, *SD* = 2 days). The children had participated in a total duration of music lessons of *M* = 17 months (*SD* = 17 months). More than half of all mothers (61.8%) and 43.6% of fathers had received musical education. The overall duration of the children’s extracurricular activities was *M* = 27 months (*SD* = 24 months). No child reported impaired hearing or color blindness. From the original sample the data of 4 participants were excluded from analysis due to technical problems (*n* = 1) or disturbances in the child’s homes (*n* = 3). This resulted in the final sample size reported above. The ethics commission of the Max Planck Society approved this study (request: 2019_25) and data were collected in 2020.

To estimate the sample size, we performed an *a priori* power analysis using G*Power version 3.1.9.4 (Faul et al., 2007, 2009) based on a study by Maier-Karius and Schwarzer (2011), which included data from 105 participants. In their study, 5- to 10-year-old children rated several melodic versions, and the reported effect size was *η*_p_^2^ = 0.39. Converted to Cohen’s *d* = 2.48 (i.e., *r* = 0.77 for comparability to our applied effect size measure), this effect is classified as extremely large (Cohen, 1988). Assuming a significance criterion of α = 0.05 and power *(1-ß)* = 0.95, the minimum sample size required for Wilcoxon signed-rank tests at this effect size is *N* = 8. Hence, the collected sample (*N* = 55) is more than sufficient to test the developmental trajectory of tonality preference and categorization.

#### 3.1.2 Apparatus, tasks, and stimuli

The apparatus and control variable measurements were identical to those used in Experiment 1. The task types were adopted for the assessment of preference and categorization. Only the musical stimuli were different, being replaced with tonal and atonal melodies that were both presented without accompaniment (i.e., in a tonality-based condition). A piano was used as an instrument timbre for all stimuli.

Since including the additional categorization task required more stimuli in our study than those used in Plantinga and Trehub (2014), we used MuseScore to generate transpositions (G major, D major, B major, and F sharp major) of the original stimulus set (C major, E major, and A major). Each child heard three transpositions strung together at random in a piano piece. Three of the seven available keys were randomly assigned to a piano piece for the preference task and another three for the categorization task. The seventh transposition was used during the training item before the categorization task. Each piano piece lasted 28.8 s (18 bars, 150 bpm, four-four time). Within the piano pieces, each transposition (motif) was repeated once in the previously randomized order (i.e., C major, G major, D major, A major, E major, B major, and F sharp major). The order of the two tasks was counterbalanced; thus, half of the sample completed the preference measurement first and the other half started with the categorization task. Moreover, the order of tonal and atonal melodies was identical per participant for all tasks, but balanced across participants. In addition, the order of pairs of puppets and within the pairs was balanced across participants. We created a third task to measure active listening preferences to gain further insight into preference development. In this task, the children selected one of the previously presented piano pieces to listen to again. However, children could shorten the duration of the stimulus presentation themselves.

#### 3.1.3 Procedure

The procedure corresponded to that used in Experiment 1, with the addition of several tasks and changes to the musical stimuli. Before beginning the experiment, children were asked to name their favorite color to check whether this was related to their ratings. No significant correlations were found when analyzing the correlation between the favorite color and the color of the puppet chosen in the experiment. For preference measurement, children listened to two consecutive piano pieces (i.e., tonal and atonal melodies) performed by different puppets. Between the two performances, the scale appeared on the screen and the rest of the procedure was explained as follows: *“Please remember how much you liked the first piece. Next, you will now hear the second piano piece.”* After listening to both piano pieces, the children rated how much they liked each one using the ice cream scoop scale (Figure 2). Categorization was tested using the same assignment paradigm as that in Experiment 1; however, the piano pieces included tonal and atonal melodies. Afterward, the children’s active listening preferences were tested by allowing them to decide whether they wanted to listen to either the tonal or atonal melody again. Finally, the selected stimulus was played twice, and the children could determine the duration each time by calling *“stop”* to end the presentation. The control variables were assessed as in Experiment 1.

### 3.2 Results

Applying the tasks evaluated in Experiment 1 offers separate measurements of preference and categorization; therefore, our analysis was divided into four parts. First, the developmental trajectory of preference for tonality was assessed in an overarching analysis of all participants. Second, the developmental trajectory of categorization was measured using the assignment paradigm from Experiment 1. Third, preference was reanalyzed considering categorization. The sample was divided into children with and without categorization ability. Split sample preference assessment was conducted to identify possible changes in ratings due to subliminal or absent perceptions of differences between stimuli. Finally, we examined the active listening preferences of the children who successfully performed the categorization in more detail.

In preliminary analysis we correlated our outcome measures with parents’ education as a proxy for socioeconomic status and found no significant correlations. Hence, for all following analyses socioeconomic status is not controlled.

#### 3.2.1 Overall preference assessment

As in Experiment 1, Wilcoxon signed-rank tests were conducted to investigate the developmental trajectory of musical preferences in 4- to 6-year-old children. In the tonality-based condition, no preferences were observed for either the tonal or atonal melody across all age groups. Overall, the participants did not prefer either melodic variant, indicating that 4- to 6-year-old children have not yet developed a preference for Western tonality.

#### 3.2.2 Development of categorization ability

To analyze how the ability to categorize musical stimuli develops, the frequencies of correct matches in the assignment paradigm were compared between age groups. Children were assigned to the “Categorization” group if both categorizations were correct within a round of two answers, otherwise they were assigned to the Non-Categorization group. The data lacked a normal distribution; therefore, chi-square tests were performed. In the tonality-based condition, chi-square tests revealed no significant differences in melody assignments o between 4- and 6-year-old children, *X*^2^ (1, *N* = 72) = 1.36, *p* = 0.243, 4- and 5-year-old children, *X*^2^ (1, *N* = 72) = 0.003, *p* = 0.958, or 5- and 6-year-old children, *X*^2^ (1, *N* = 76) = 1.32, *p* = 0.251. This suggests that the ability to categorize tonal changes does not significantly improve between 4 to 6 years of age. Nevertheless, the number of correct assignments exceeded false assignments for 6-year-old children, and the percentage of correct assignments increased between the ages of 4 (44.12%) and 6 (57.89%) years.

#### 3.2.3 Split preference assessment

Categorization ability is still developing between the ages of 4 and 6 years and should theoretically precede the development of any systematic preferences. Thus, children who cannot yet successfully categorize tonal and atonal melodies should not show a preference toward either category. This offers a helpful approach to test our method. If our methods for separately assessing categorization and preference are valid in this age group, then we should be able to see significant systematic preferences for tonal melodies only in children who perform well on the categorization task. To assess this, we reanalyzed participants’ preference ratings using Wilcoxon signed-rank tests and divided our sample into children who successfully categorized and those who did not. The classification as successful categorization was based on the correct assignment of both stimulus variants in one round. We discovered, as a complement to the overall preference analysis, that only 6-year-old children who successfully categorized the tonal and atonal melodies showed a preference for the tonal melody. Further results of split preference assessment are summarized in Table 3.

**Table 3:** Wilcoxon signed-rank tests for tonality preferences across age groups.

Melody	Age											
	4 years				5 years				6 years			
	*z*	*p*	*r*	*95% CI*	*z*	*p*	*r*	*95% CI*	*z*	*p*	*r*	*95% CI*
Tonal against												
Atonal	−0.50	0.536	0.16	[−2.99, 1.50]	−0.50	0.454	0.21	[−1.50, 0.99]	−1.00	0.100	0.43	[−1.50, −0.00]

**Figure 5:**
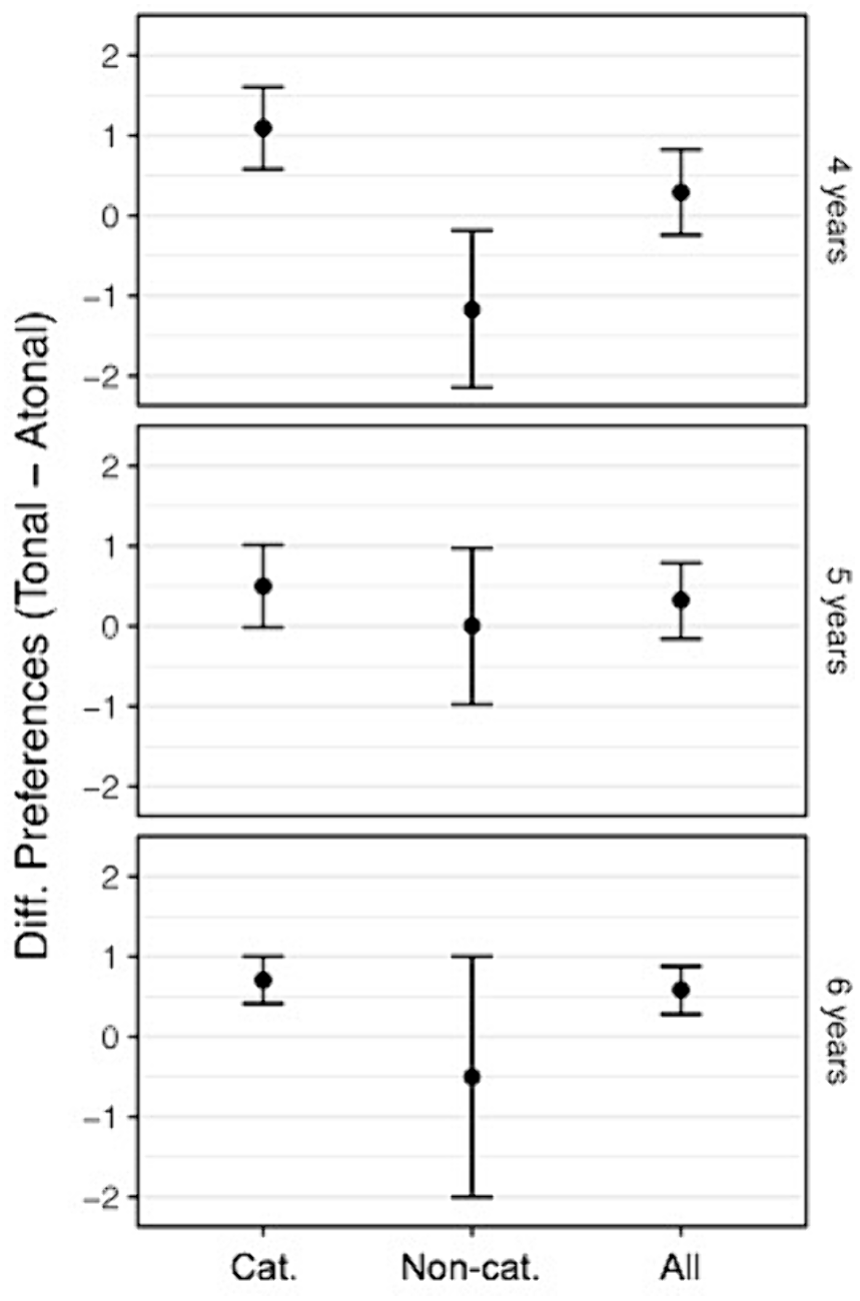
Preference difference scores and 95% confidence intervals. Preference difference scores for 4- to 6-year-old children who rated piano pieces either with a tonal or an atonal version of the melody. Dividing the total sample (all) into participants with categorization abilities (cat.) and without (non-cat.) highlights that preferences differ based on categorization ability and that there is more variation in the ratings of participants without categorization ability.

Preference scores were converted to difference scores (i.e., differences between the ratings for tonal and atonal melodies) and are presented in Figure 5.

#### 3.2.4 Active listening preference

Based on the split preference analysis, we examined the active listening preferences of children with categorization abilities. The frequency of selecting tonal melodies for re-listening increased from 45.5% in 4-year-old children up to 64.7% in 6-year-old children. However, no significant differences were found in stimulus selection between age groups. For analyzing listening duration in the re-listening task, Mann–Whitney *U* tests were performed to evaluate whether chosen melodic versions differed. Children listened to their chosen version twice, making it possible to calculate a cumulative listening duration. Overall, no significant differences were observed in any age group. Thus, the melodic version children selected made no difference in re-listening duration. Overlapping with split preference analysis, active listening preference based on selection frequency showed a beginning of a shift toward a tonal preference.

### 3.3 Discussion

Considering the entire sample, we observed that categorization ability for tonal and atonal melodies improved with age; however, the children did not yet show any tonality preference. However, 6-year-old children who could already categorize between stimuli did show a preference; therefore, the assumption of no preference is misleading. Children without categorization ability showed no preference, suggesting that categorization ability might proceed preference development. The results on preference for active listening support those of preference measurement by scale with respect to that preference for tonality emerges with increasing age.

## 4 Experiment 3: development of consonance preference and categorization

Based on findings from Experiments 1 and 2, our aim in Experiment 2 was to gain insights into the development of preferences and categorization ability for consonance and dissonance. As in Experiment 2, we chose existing stimuli from Plantinga and Trehub (2014) and investigated 4- to 6-year-old children.

### 4.1 Method

#### 4.1.1 Participants

The final sample comprised 112 children (59 female) aged 4 to 6 years (*M* = 66 months, *SD* = 10 months). Participants were divided into three age groups: 4-year-old (*n* = 34), 5-year-old (*n* = 39), and 6-year-old (*n* = 39) children. Recruitment and rewards for participation were identical to those in Experiments 1 and 2. In the largest proportion of the sample, both parents had a university degree (39.3%); in 33.9% neither parent had a university degree, and in 26.8% one parent had a university degree. Monthly family income ranged from under 2,000–3,000€ (17.9%) to more than 5,000€ (27.6%). Most parents reported their monthly income as 4,000–5,000€ (27.7%), followed by 3,000–4,000€ (26.8%). All children in the sample were born in Germany and spoke German as their native language. Children listened to music in their home environment for an average of 10 h per week (*SD* = 10). Among parents, 75.0% reported that they made music with their child every week (*M* = 3 days, *SD* = 2 days). The Overall duration of music lessons that children attended was *M* = 20 months (*SD* = 10). Over half of all mothers (54.5%) and 40.2% of fathers had musical training. The average duration of extracurricular activities of the participating children was 28 months (*SD* = 27 months). No children reported impaired hearing or color blindness. Data were collected in 2020 and the study was previously approved by the ethics commission of the Max Planck Society (request: 2019_25). From the original sample the data for eight participants were excluded from analysis due to technical problems (*n* = 2), communication problems, (*n* = 4), or disturbances in the child’s home (*n* = 2). This resulted in the final sample reported above.

We determined the sample size using an *a priori* power analysis with G*Power version 3.1.9.4 (Faul et al., 2007, 2009) based on a study by Weiss et al. (2020), which examined preferences for consonant and dissonant stimuli of 40 participants. The indicated effect size of this study was *η_p_^2^* = 0.67, corresponding to *r* = 0.82 (for comparability to our applied effect size measure) and Cohen’s *d* = 2.84, which is considered extremely large (Cohen, 1998). The minimum sample size required for Wilcoxon signed-rank tests at this effect size, with a significance criterion of α = 0.05 and a power *(1-ß)* of 0.95, is *N* = 5. Since Experiment 3 contains two separate conditions and participants were randomly assigned to only one, the minimum sample was required for both conditions, and thus is *N* = 10. Thus, the sample of 112 participants in Experiment 3 is more than adequate to test the development of consonance preference and categorization.

#### 4.1.2 Apparatus and stimuli

**Figure 6:**
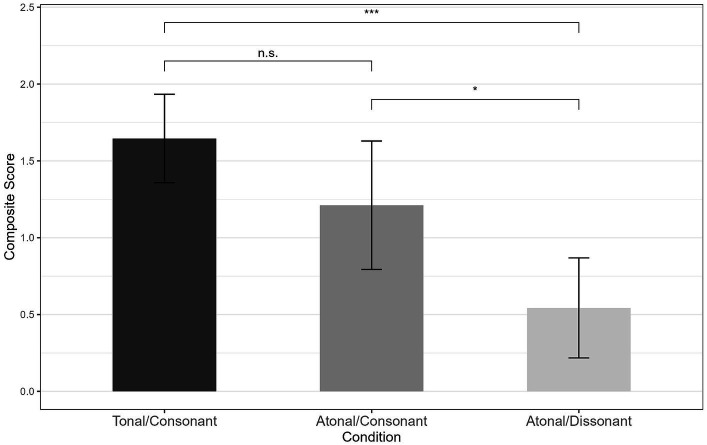
Mean composite scores of the extent of consonance and standard deviations. Mean composite scores were calculated for slight-difference-based condition 1 (tonal melodies with consonant harmonies vs. tonal melodies with dissonant harmonies) and large-difference-based condition 2 (tonal melodies with consonant harmonies vs. atonal melodies with dissonant harmonies). *p < 0.05. ***p < 0.001.

The apparatus and control variable measurements were identical to those in Experiments 1 and 2. Consonance preference and perception were measured in a *slight-difference-based condition* (Condition 1; tonal and atonal melodies from Experiment 2 accompanied by consonant harmonies) and *large-difference-based condition* (Condition 2; tonal melodies with consonant harmonies and atonal melodies with dissonant harmonies). Each child participated in only one condition. Before the experiment began, stimuli were analyzed with respect to their degree of consonance using the R package *incon* (Harrison and Pearce, 2020). The analysis allowed for the amount of consonance in simultaneous stimuli to be predicted using a multidimensional model. Figure 6 presents the composite scores, which are non-standardized regression coefficients representing the degree of consonance. Composite scores for tonal melodies with consonant harmonies (*Mdn* = 1.88) did not differ significantly from those for the atonal melodies with consonant harmonies (*Mdn* = 1.22) in Condition 1, *U* = 34.00, *z* = −1.22, *p* = 0.247, *r* = 0.27. However, in Condition 2, composite scores of tonal melodies with consonant harmonies (*Mdn* = 1.88) differed significantly from those for the atonal melodies with dissonant harmonies (*Mdn* = 0.39), *U* = 6.00, *z* = −3.33, *p* < 0.0001, *r* = 0.74. Moreover, composite scores of atonal melodies with consonant harmonies in Condition 1 (*Mdn* = 1.22) differed significantly from those for atonal melodies with dissonant harmonies in Condition 2 (*Mdn* = 0.39), *U* = 19.00, *z* = −2.34, *p* = 0.019, *r* = 0.52.

Although the stimuli used in this study were the same as in Plantinga and Trehub (2014), the evaluation of degree of consonance and dissonance differed slightly. This is due to the different evaluation methods. In Plantinga and Trehub (2014) adults rated the stimuli, whereas in this study the stimuli were rated with the R package incon. Human adults rated tonal melodies with consonant harmonies in the large-difference-based condition higher than those in the slight-difference-based condition, although the stimuli and composite scores were the same. Otherwise, we replicated the absence of any significant difference between the stimuli in Condition 1. Plantinga and Trehub (2014) overall concept was also possible to support, as the extent of consonance in the stimuli with atonal melodies decreased significantly between Condition 1 and Condition 2 (Figure 6). More detailed information about the progression of the amount of consonance within all stimuli is shown in Supplementary Figure S1. Despite the small discrepancy we decided to use these stimuli because it allowed us to gather information about preference development and provided comparability with previous studies.

#### 4.1.3 Procedure

The procedure was consistent with that used in Experiment 2.

### 4.2 Results

In preliminary analysis we correlated our outcome measures with parents’ education as a proxy for socioeconomic status and found no significant correlations. Hence, for all following analyses socioeconomic status is not controlled.

#### 4.2.1 Overall preference assessment

**Figure 7:**
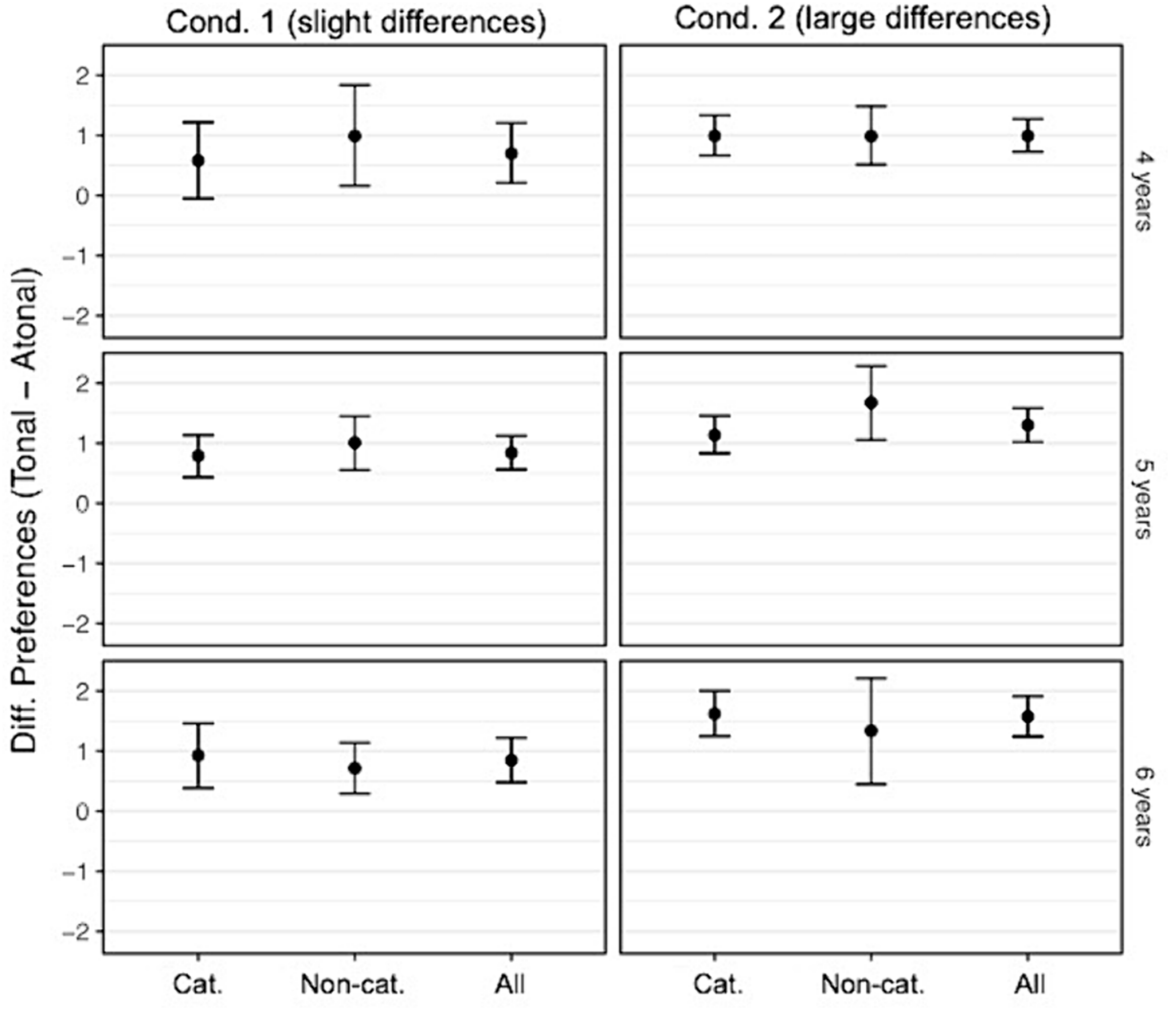
Preference difference scores and 95% confidence Intervals for Conditions 1 and 2. Preference difference scores for 4- to 6-year-old children who rated accompanied piano pieces either with a tonal or an atonal version of the melody. Dividing the total sample (all) into participants with categorization abilities (cat.) and without (non-cat.) highlights that preferences differ based on categorization ability and that there is more variation in the ratings of participants without categorization ability.

We performed Wilcoxon signed-rank tests to investigate the developmental trajectory of consonance preference in 4- to 6-year-old children, given the absence of normally distributed data. The preference difference values from the overall analysis are shown in the right column of each panel in Figure 6. For the stimuli in Condition 1, a significant preference was observed starting from the age of 5 years. Specifically, 5-year-old children preferred the tonal melodies with consonant harmonies over atonal melodies with consonant harmonies, *z* = −1.49, *p* = 0.009, *r* = 0.60, 95% CI [−1.99, 0.49], as did 6-year-old children, *z* = −2.04, *p* = 0.041, *r* = 0.46, 95% CI [−2.50, −0.000073]. In Condition 2, in which the musical stimuli differed the most, all age groups showed a preference for tonal melodies with consonant harmonies. Specifically, 4-year-old (*z* = −1.49, *p* = 0.005, *r* = 0.71, 95% CI [−2.00, −0.99]), 5-year-old (*z* = −1.50, *p* = 0.001, *r* = 0.77, 95% CI [−2.00, −0.99]), and 6-year-old children (*z* = −1.99, *p* = 0.001, *r* = 0.79, 95% CI [−2.50, −1.00]) rated tonal melodies with consonant harmonies significantly higher than atonal melodies with dissonant harmonies. In particular, in Condition 2, the preference difference values showed that atonal melodies with dissonant harmonies were decreasingly liked with increasing age, as the ratings show a positive trend toward a preference for tonal melody with consonant harmony (Figure 7). Overall, 4- to 6-year-old children preferred tonal melodies with consonant harmonies if a large difference existed between musical stimuli (Condition 2). However, if musical stimuli were more similar, as was the case in Condition 1 given the similar harmonical accompaniment, preference started later, at 5 years of age.

#### 4.2.2 Development of categorization ability

In Condition 1, no significant changes appeared between age groups. Categorization did not change significantly between 4- and 6-year-old children, *X*^2^ (1, *N* = 74) = 0.22, *p* = 0.641, 4- and 5-year-old children, *X*^2^ (1, *N* = 72) = 0.001, *p* = 0.979, or 5- and 6-year-old children, *X*^2^ (1, *N* = 78) = 0.21, *p* = 0.651. However, a clear developmental trajectory was observed in Condition 2. The number of correct assignments increased with age. Specifically, 6-year-old children assigned significantly more piano pieces to the correct puppet than did 4-year-old children, *X*^2^ (1, *N* = 72) = 6.82, *p* < 0.01. Moreover, a marginally significant difference was found between 4- and 5-year-old children, with 5-old children having slightly more correct responses, *X*^2^ (1, *N* = 74) = 3.82, *p* = 0.051. Responses were similar between 5- and 6-year-old children (no significant difference, *X*^2^ (1, *N* = 78) = 0.54, *p* = 0.464). Taken together, children’s categorization was better with larger differences between consonant and dissonant stimuli. Response frequencies for both conditions are listed in Table 4.

**Table 4:** Responses across all conditions in the assignment paradigm.

Age	Slight-difference-based Condition 1		Large-difference-based Condition 2	
	Correct		Correct	
	*n*	%	*n*	%
4 years	18	52.94	11	32.35
5 years	20	52.63	22	55.00
6 years	19	47.50	24	63.16

#### 4.2.3 Split preference assessment

In Condition 1, we found that the preference for tonal melodies with consonant harmonies among 5-year-old children occurred for those who could categorize. However, this could not be supported in 6-year-old children when the sample was divided, because neither 6-year-olds with (nor without categorization abilities showed any preference). Table 5 shows the results of all the comparisons carried out for Condition 1 and Condition 2 for children with successful categorization.

**Table 5:** Wilcoxon signed-rank tests for consonance preferences across age groups.

Stimuli	Age											
	4 years				5 years				6 years			
	*z*	*p*	*r*	*95% CI*	*z*	*p*	*r*	*95% CI*	*z*	*p*	*r*	*95% CI*
Condition 1 TM with CH against												
AM with CH	−0.50	0.410	0.24	[−2.50, 1.00]	−1.00	0.004	0.54	[−2.00, 0.00]	−1.22	0.154	0.41	[−3.99, 0.49]
Condition 2 TM with CH against												
AM with DH	−1.50	0.033	0.76	[−2.00, −1.00]	−1.00	0.004	0.82	[−2.00, −1.00]	−1.50	0.002	0.79	[−2.50, −1.00]

In Condition 2, the results from the overall analysis could again be extended to show that only 4-year-olds, 5-year-olds, and 6-year-olds who were able to categorize stimuli showed a preference toward tonal melodies with consonant harmonies. Preference difference scores (e.g., differences between ratings of tonal melodies with consonant harmonies and atonal melodies with consonant harmonies in Condition 1) are shown in Figure 7.

#### 4.2.4 Active listening preference

We further analyzed the active listening preferences of children with categorization abilities in Conditions 1 and 2. Notably, in Condition 1, the frequency of choosing tonal melodies with consonant harmonies for re-listening increased from 50.0% in 4-year-old children to 78.6% in 5-year-old children. Otherwise, no significant differences were observed in listening duration or stimulus selection between age groups. In Condition 2, an above-average frequency of selecting tonal melodies with consonant harmonies was observed across age groups. Therefore, no significant differences in stimulus selection were found between age groups. Children as young as 4 years of age selected tonal melodies with consonant harmonies to hear again 70% of the time. No significant differences were found for listening duration in Condition 2.

### 4.3 Discussion

Preferences for and perceptions of consonance versus dissonance have long been conflated in studies on perceptual consonance preference, while insights into their development during childhood have been rare. We have addressed these gaps by developing a child-friendly method for separately assessing categorization ability and preferences and exploring how each develops between the ages of 4 to 6 years.

Analyzing the data from all participants together, we observed that the ability to categorize between consonance and dissonance improves with age, followed by a developing consonance preference in 4- to 6-year-old children. For harmonized stimuli with slight differences, a preference for tonal melodies with consonant harmonies began at the age of 5 years (Condition 1). Slight support for this also comes from descriptive results on active listening preference, where we observed a strong increase in the selection of tonal melodies with consonant harmonies from the age of 5 years (this increase was not statistically significant, only descriptively observable). Earlier onset of preference at the age of 4 years occurred with large differences between stimuli (Condition 2). However, the assumption of preferences for entire age groups at this stage is deceptive. Crucially, only children who could also appropriately categorize stimuli showed a preference, suggesting that categorization might precede preference. This indicates why perception in the form of categorization needs to be considered in preference analysis. Moreover, preference and perception should not be treated equally. Categorization ability is still developing between 4 to 6 years of age, as shown by earlier and better categorization of strongly over weakly contrasting consonant and dissonant stimuli. If the tonal melodies were presented with consonant harmonies and strong contrasts to the dissonant version, then preferences began as early as 4 years of age. Additional support for this preference is provided by measures of active listening preference measurement, as 4- to 6-year-old children with successful categorization preferred tonal melodies with consonant harmonies for re-listening.

## 5 General discussion

The aim of our study was to develop a new methodological approach to assess consonance perception and preference, and to explore the development of preferences for tonality and consonance in childhood. A graphical summary of our study is shown in Supplementary Figure S2. In Experiment 1, we evaluated the new methodological approach with 4- to 6-year-old children. All three age groups were able to indicate their preferences for food and puppet-drawn pictures. The evaluation of the categorization paradigm showed a developmental trajectory in which the number of correct categorizations increased with age. Therefore, the methods evaluated in Experiment 1 were used in Experiment 2 (tonality) and Experiment 3 (consonance) to explore the developmental trajectory of preferences and categorization ability in 4- to 6-year-old children.

In Experiment 2 we found that the ability to categorize tonal and atonal melodies develops between 4 and 6 years of age. No significant changes were observed between the age groups, with 6-year-olds making more correct than incorrect categorizations. The most striking finding was that dissociating perception (i.e., dividing the children into groups with and without categorization ability) led to different results. Contrary to the overall preference analysis (no preference in any age group), we found a preference for tonal melodies in 6-year-old children who could differentiate between tonal and atonal melodies. We also observed an increase in active listening tonal melodies with increasing age in children who showed categorization skills.

In Experiment 3, categorization analysis revealed developmental trends in 4- to 6-year-old children. Although almost no change was observed in Condition 1 (slight differences between stimuli), the age progression became clearer in Condition 2 with large differences between stimuli. In particular, 6-year-old children categorized significantly better than 4-year-old children, and categorization accuracy increased with age. Further preference analysis of Condition 1 showed that tonal melodies with consonant harmonies were preferred at the age of 5. Taking into account categorization ability, this preference was only evident for children who could correctly categorize tonal melodies with consonant harmonies and atonal melodies with consonant harmonies. At the same time, the active listening preference for tonal melodies with consonant harmonies increased. A preference analysis of Condition 2 showed that the preference for tonal melodies with consonant harmonies started even earlier, at 4 years of age, and remained for 5- and 6-year-old children. In addition, the preference for tonal melodies with consonant harmonies was found only in children with good categorization skills from the age of 4, and all age groups chose these stimuli for re-listening.

Our new method, evaluated in Experiment 1, provides a powerful approach to measuring preferences and perceptions (i.e., categorization) separately in 4- to 6-year-old children. Children in all three age groups used the entire scale to indicate their preferences. However, future studies should take into account the working memory load of the categorization task. It remains unclear whether reducing the visual input during the task to only one puppet and asking children of the same age group to judge performance (e.g., did the puppet make mistakes? Did the puppet play the same piece?) would result in younger children showing better categorization skills. In addition, it might be interesting in future experiments not only to look at the influence of preferred color, as we did, but also to look for cross-modal correspondences (i.e., if tonality corresponds to a particular color, see Spence and Di Stefano (2022)).

The focus on the development of tonality, the preference for tonal melody in 6-year-old children, and the improvement in tonal perception between the ages of 4 and 6 support the idea that tonality is a complex musical phenomenon that is not fully developed by the age of 6 (Schwarzer et al., 1993). Although early developing pitch-related skills (e.g., detection of contour-violating changes; Trehub et al., 1984, 1985, 1990; Morrongiello et al., 1985) are present in infancy, our findings support previous research suggesting that enculturation into Western tonality is a tonal skill that develops in late childhood (Trainor and Hannon, 2013; Corrigall and Trainor, 2014). Consistent with Maier-Karius and Schwarzer (2011), our results indicate a tonality preference at 6 years of age. Furthermore, our findings of an increase in categorization ability with age are consistent with a more refined distinction emerging from previous research, and complement previous findings by providing a potential underlying mechanism for this phenomenon (e.g., Maier-Karius and Schwarzer, 2011).

With respect to consonance, no preference was found in the slight difference-based condition for both 4- and 6-year-old children in this study or for 6-month-old infants in a previous study (Plantinga and Trehub, 2014). As pointed out earlier, this condition is the one with the higher cognitive load and is therefore more difficult to solve. Categorization, as an underlying mechanism of consonance preference, was found to be still developing between 4 and 6 years of age. On closer inspection, categorization was shown to become more accurate with age, as children gained more experience in perceiving consonance and dissonance. These findings are consistent with observations of both a progressive “tuning” of musical perception to the surrounding musical culture (Trehub et al., 1990; Lynch and Eilers, 1992) and a shift toward culture-specific strategies (Trehub et al., 1997) for perceiving consonance and dissonance as distinct musical phenomena. The preference for consonance is stimulus dependent in early childhood and requires strong contrasts such as those in Condition 2. Taken together, these findings highlight that preference for consonance in Western adults is acquired during childhood when they are exposed to Western musical culture, consistent with the claim that aspects of perception are shaped by cultural experiences (Trehub et al., 2015).

In contrast to Plantinga and Trehub (2014), we found a preference for tonal melodies with consonant harmonies in Condition 2 across all age groups, despite using the same musical stimuli as in that previous study. This is notable because we found a preference from 4 years of age, which is earlier than reported by Valentine (2015) and Weiss et al. (2020). Future research should explore this age discrepancy in more detail, as earlier onset may be due to methodological differences.

Our findings highlight developmental changes in consonance preference and perception in 4- to 6-year-old children. Interestingly, preferences depended on the amount of difference in the musical stimuli, appearing earlier for stimuli with large differences, which is in accordance with the assumed cognitive load of the tasks. One interpretation of this is that signs of preferential consonance perception in infancy change during musical enculturation in childhood and manifest as a preference for consonance in Western-socialized adults. Two additional findings support this explanation. First, the results on tonality provide support by indicating a similar developmental trajectory. Children indicated a preference for tonal melody at the age of 6, presumably influenced by their exposure to Western tonality. As expected, the preference for tonality increases during childhood, and musical enculturation takes place during this period. Secondly, categorization ability is still developing at 6 years of age and may even improve afterwards. This notion is supported by our finding that consonance was categorized more accurately for stimuli based on large differences. As children’s perceptions of consonant and dissonant sound categories develop, they first learn to reliably categorize highly contrasting sounds (i.e., very consonant and very dissonant sounds). Over time, children become better at categorizing weakly contrasting sounds. Finally, we found that categorization influences the development of preferences. Crucially, only children with categorization skills showed preferences, and categorization appears to be a prerequisite for consonance preference in 4- to 6-year-old children.

Taken together, our findings suggest that tonality and consonance preferences are complex musical skills that change during enculturation between the ages of 4 and 6. Notably, both preferences were found to occur only in children with categorization abilities, thus sharing an underlying perceptual mechanism. To our knowledge, this study is the first to measure preference and perception separately in 4- to 6-year-old children, allowing us to explore this underlying mechanism. Nevertheless, equating tonality preference with consonance preference should be avoided. One reason is that consonance preference in our study was stimulus dependent and emerged at 4 years of age, when large differences between consonance and dissonance were present. However, the first indication of tonality preference was observed later in 6-year-old children. Future work needs to follow the development of preferences for tonality and consonance to explore whether categorization ability remains a common mechanism with age or to what extent the two preferences diverge. It may be interesting to consider that the reported preference for consonance seems to be based on a different form of judgment in infants than in children. Infants show this preference, then it seems to disappear, and then re-emerges later in childhood, but on a different basis. This kind of developmental trajectory (disappearing and re-emerging in different form) might be similar to the developments found in false belief tasks. This development can be understood in the framework of the Representational Redescription Model (Karmiloff-Smith, 1992), in which development means getting increased conscious access to knowledge which is already in the cognitive system, however, in a primitive or rudimentary form.

Our study has a number of methodological limitations. The first concerns the musical stimuli, which were categorical and artificial. No conclusions can be drawn about a preference for consonance in real musical pieces or about a gradual understanding of consonance and dissonance. Therefore, replications with existing stimuli are necessary to identify possible preferences, which can then be tested with more naturalistic and new stimuli, such as real musical pieces. A second potential limitation may be the categorization paradigm with two puppets. Solving the categorization paradigm required cognitive representation of both puppets and their performances. This cognitive load may have been too much for the working memory of 4- to 6-year-olds; thus, we may have underestimated the children’s true categorization abilities.

Regarding the use of puppets in general, a recent special issue on the use of puppets in developmental research raised concerns about the comparability of children’s responses or reasoning about puppets and real people, particularly in the context of theory of mind tasks (Packer and Moreno-Dulcey, 2022). However, for this type of task, children seem to perform essentially the same in tasks with puppets and real people, as shown in a meta-analysis (Yu and Wellmann, 2022). For our experiments, it seems reasonable to mention that we do not have the categorization as well as the preference ratings for real person performances, but this may not be so important here as it should not matter so much who is performing for this type of task. All in all, using puppets may provide the basis for less socially influenced responses by the children. An adult instead of a puppet might cause a pressure for socially desirable answers given by the child. The third methodological limitation relates to online data collection. As the participants’ home environment was less controllable and more natural than the laboratory environments used in previous studies, we had to exclude more data from our analysis than in previous studies. These limitations highlight the difficulty of conducting age-appropriate preference measures that separate preferences from perceptions online. As we focused on Western-socialized 4- to 6-year-old children, cross-cultural comparisons are needed to clarify developmental trajectories in other cultures. Furthermore, comparisons with older age groups are needed to analyze whether tonality or consonance preferences continue to stabilize and at what point this is comparable to that of Western adults, especially with respect to consonance perception.

## 6 Conclusion

Despite the limitations of the study, our findings suggest, as a theoretical implication, that developmental changes in tonality and consonance preferences and perceptions occur between 4 and 6 years of age. Our study adds to the understanding that the complex musical skill preferences for consonance and tonality in Western-socialized children are acquired through enculturation. Furthermore, we show that perceived differences (between tonality and atonality or consonance and dissonance) might be a crucial underlying factor in the development of preferences. In conclusion, the present study advances our understanding of tonality and consonance preferences and perceptions. In particular, the results show why preferences and perceptions should not be conflated. We show that the overall analysis overestimates preferences in 4- to 6-year-old children, that the results of our split preference analysis differ significantly from this, and that preferences for consonance and tonality are only evident in children with categorization abilities. We hope that our study can provide a basis for further research into the factors underlying consonance preference and its development throughout childhood.

## Data availability statement

The raw data supporting the conclusions of this article will be made available by the authors, without undue reservation.

## Ethics statement

The studies involving humans were approved by Ethics Council of the Max Planck Society. The studies were conducted in accordance with the local legislation and institutional requirements. Written informed consent for participation in this study was provided by the participants’ legal guardians/next of kin.

## Author contributions

JW: Conceptualization, Data curation, Formal analysis, Investigation, Methodology, Visualization, Writing – original draft, Writing – review & editing. CR: Conceptualization, Supervision, Writing – review & editing. FD: Conceptualization, Supervision, Writing – review & editing.

## Footnotes

^1^We conducted multiple comparisons. The bonferroni-corrected level would be 0.05/3 = 0.017.
