# Corrigendum: Development of tonality and consonance categorization ability and preferences in 4- to 6-year-old children

**DOI:** 10.3389/fpsyg.2024.1460303

**Published:** 2024-09-30

**Authors:** Johanna Karoline Will, Christina Roeske, Franziska Degé

**Affiliations:** Max Planck Society, Max Planck Institute for Empirical Aesthetics, Music Department, Frankfurt, Germany

**Keywords:** development, preference, perception, tonality, consonance

In the published article, there was a citation error. The incorrect sentence previously stated, “A similar endeavor for infants and younger children has been undertaken by Di Stefano et al. (2022)”. Instead of “Di Stefano et al. (2022)”, it should be “Di Stefano et al. (2017)”. The citation has now been inserted in **Introduction**, *Paragraph 6*, and the correct sentence should read, “A similar endeavor for infants and younger children has been undertaken by Di Stefano et al. (2017).”

The authors apologize for this error and state that this does not change the scientific conclusions of the article in any way. The original article has been updated.
