# Supplementary material for: Development of tonality and consonance categorization ability and preferences in 4- to 6-year-old children
Source: Front Psychol. 2024 Jun 17;15:1270114. doi: 10.3389/fpsyg.2024.1270114 (PMC11336827; doi:10.3389/fpsyg.2024.1270114)
Supplement: Supplementary file 1 [file Data_Sheet_1.docx]

Supplementary Material

The Development of Complex Musical Skills:

Categorization and Preference of Tonality and Consonance in 4- and 6-year-old Children

Johanna Karoline Will^1^, Christina Roeske^1^, Franziska Degé^1*^

^1^ Max Planck Institute for Empirical Aesthetics, Music Department, Max Planck Society, Frankfurt/M., Germany

*** Correspondence:**Franziska Degé
Franziska.Degé@ae.mpg.de

# Supplementary Figures

**Supplemental Figure 1**

*Progression of Consonance Scores for Condition 1 and Condition 2 of Experiment 3*

*Note.* Tonal melody with consonant harmonies (Ton./Cons.) were used in Condition 1 and Condition 2 of Experiment 3. Atonal melodies with consonant harmonies (Aton./Cons.) were part of Condition 1 to create slight difference between stimuli by changing only the melody. To implement large differences between stimuli in Condition 2, tonal melody with consonant harmonies (Ton./Cons.) compared to atonal melodies with dissonant harmonies (Aton./Diss.). In the progression, the amount of consonance (consonance scores) repeatedly overlap due to the consonant accompaniment of Ton./Cons. and a. Aton./Cons.. At position 2-2, a resolution in the piano piece causes the Aton./Diss. stimuli to have the highest degree of consonance for a short time. At all other positions, however, the progression analysis clearly shows that the Aton./Diss. stimuli have the lowest degree of consonance.

**Supplemental Figure 2**

*Graphical Abstract*

*
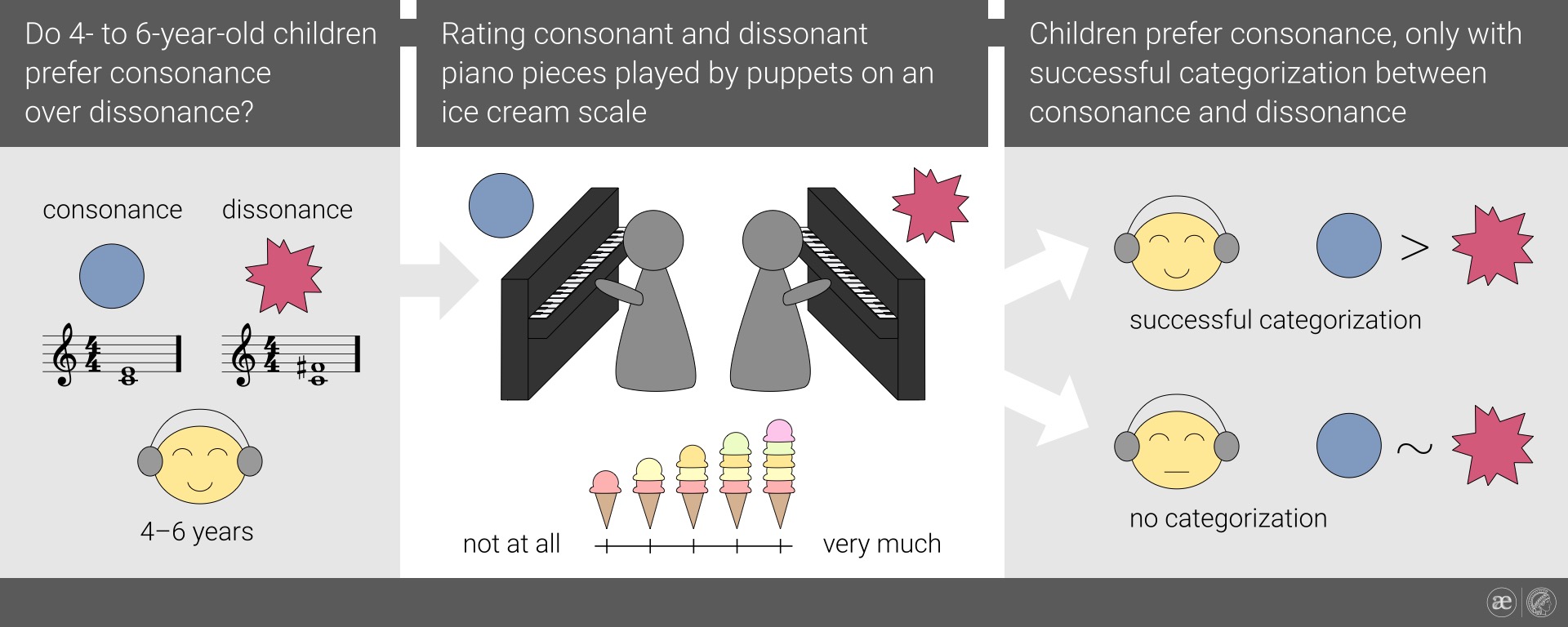
*
